# How to Keep Warm

**Published:** 1881-03

**Authors:** 


### HOW TO KEEP WARM.

In very cold weather most people have sense enough t build good fires and wear their thickest clothing; few, however, seem to know that physical warmth is created in the body itself, and all that fires or clothing can do is to prevent the warmth being seized too rapidly by the surrounding air. The best preparation for a comfortable day in very cold weather is to eat a generous breakfast, in which there shall be plenty of meat. There is far more warmth in an ounce of cold meat than in a pint of hot coffee, although the latter is to thousands of people the principal feature of the morning meal. A good appetite is necessary to a full breakfast, and it generally can be had by a five-minute walk out of doors or a few minutes of light exercise in a freshly-aired room—exercise such as the most delicate woman or child can indulge in without injury. Physical cleanliness, making free perspiration possible, is absolutely necessary to comfort in cold weather, and it can be attained, in spite of freezing cold bath rooms, by people who care enough for it to take extra trouble with a small quantity of water in a warm room. A glass of ardent liquor is a wretched preventive of cold. It will quicken the circulation for a few moments and diminish it for an hour after. The bulk in bread of a glass of beer is more warming than the liquor and only costs a quarter as much; the same comparison may be made between spirits and meat. It is almost impossible for a person who sits indoors all day to remain warm, but a few minutes out of doors, just long enough to have the system affected enough by the cold to rouse its powers of resistance, will insure a comfortable thereafter if the house is fairly tight. It will be noticed that the lady who does her own marketing, and the man who walks from his house to his place of business, are the last to complain of the cold. If the above suggestions are acted upon and supplemented by an ample midday meal, no matter how plain, the weather's terrors will soon be forgotten.—*New York Herald.*

---

Such as thy words are, such will thy affections be esteemed; and such will be thy deeds as thy affections, and such thy life as thy deeds.—*Socrates.*

He that opposes his own judgment against the current of the times ought to be backed with unanswerable truth ; and he that has truth on his side is a fool, as well as a coward, if he is afraid to own it, because of the multitude of other men's opinions.

